# An Inquiry into the Nature and Pathology of Granular Disease of the Kidney, &c.

**Published:** 1842-04-01

**Authors:** 


### An Inquiry into the Nature and Pathology of Granular Disease of the Kidney, and its Mode of Action in producing Albuminous Urine. By *George Robinson.* London, John Churchill, pp. 79.

The leading object of Mr. Robinson is expressed in the following passage of his preface.

"It is true that the probability of the congestive or inflammatory character of the disease has been alluded to in different works, but to my knowledge no attempt has yet been made to reconcile that view with all the numerous varieties and irregularities that have been observed in different cases: whatever credit, therefore, may attach to the person who first endeavoured to accomplish this end, I may perhaps be permitted to claim." vii.

In his first chapter, Mr. Robinson, after alluding to the contradictory opinions on the nature of the granular disease which have obtained, proceeds to state his own opinions thus:—

"From the limited extent of the granular deposit and other peculiarities distinguishing it from diseases of constitutional origin, it seems fair to conclude that it is a local affection; and from certain facts, to be hereafter mentioned, and from a general observation of its symptoms, causes, morbid appearances, &c., and a comparison of them with those noticed in inflammation of other parts, I have been led to think that the acute stage of Dr. Christison is simply acute nephritis, and that all the varieties of morbid appearances occurring in the chronic stage, may be considered as resulting from so many different degrees in intensity and duration of chronic inflammation of the kidney.

All authors seem to have agreed that the acute stage or form is a congestive, if not an inflammatory disease of an acute character, and have regulated their treatment accordingly. M. Rayer considers it to be a variety or modification of inflammation, and calls it albuminous nephritis. Dr. Christison says, 'that it is not improbable that some of the instances, where a dark, flabby, enlarged state of the kidneys has been found in connexion with coagulability of the urine, &c. have been nothing more than cases of ordinary inflammation, or pure nephritis:' whilst M. Solon conceives that true nephritis is quite another disease; but his distinctions between them are destroyed by Dr. Christison, who, however, so far from asserting their identity adopts an arrangement which recognises the existence of an acute congestion or inflammation of the kidney distinct from nephritis; and moreover says, 'In this city (Edinburgh) we have few opportunities of ascertaining the diagnosis between nephritis and granular degeneration, as the former is an extremely rare disease.'

This opinion, as to the rare occurrence in this country of idiopathic nephritis, is held by most physicians, at least very few have recognised many cases of it, and the majority say they have not met with a case of acute idiopathic inflammation affecting the kidney. Why organs performing so important a function, and so largely supplied with blood, which are especially subject to irregularity in their amount of action, and to sudden and extreme increase of vascularity from their sympathy with, and antagonism in function to the skin, should be so remarkably exempt from inflammatory action, when we find all the other important viscera of the chest and abdomen so frequently inflamed from the operation of causes which must act with at least equal power on the kidneys, is a mystery which has never been satisfactorily explained. If all the cases of the acute form of granular disease be considered as instances of idiopathic nephritis, then the latter disease will be found to be at least as frequent in its occurrence as most other acute visceral inflammations." 10.

We confess that we have long taken a very similar view. We have entertained and expressed the opinion, that the granular disease of the kidney is essentially inflammatory or congestive, analogy as well as positive facts seeming to lend support to this conclusion. The granular disease is a common consequence of stricture of the urethra, and we can understand why, being an inflammatory affection, it should be so. We apprehend that, ultimately, pathologists will come to view the matter in this light.

The second chapter contains a comparison of the symptoms of the acute stage or form of granular disease, with those of acute nephritis. In this stage, says Mr. Robinson, the symptoms are rigors and other usual signs of inflammatory fever; pain in the loins, more or less increased on pressure; pain across the pit of the stomach; nausea, and vomiting, with an irritable or even inflammatory condition of the bladder; and so far they precisely correspond with those of nephritis. But there are two additional ones which are certainly not mentioned by authors as occurring in the latter disease, viz. the frequent co-existence of acute inflammatory anasarca, particularly affecting the hands and face, and a scanty, bloody, highly albuminous condition of the urine. With respect to the former symptom, as idiopathic nephritis is considered to be so rare a disease in this country, it is possible that late writers may not have recognised a sufficient number of cases to enable them to decide whether this anasarca is or is not occasionally present in that disease. At any rate, as it is not an invariable and constant accompaniment of the acute stage; so, supposing the latter to be but nephritis, the presence or absence of this symptom will not of itself be sufficient to decide the question in any particular case.

Mr. Robinson seems to aver that albumen does occur in the urine in nephritis, and draws from the assertion support to his argument. In the *chronic stage* the symptoms in many respects are similar to those of the acute, but diminished in intensity so far as the local affection is indicated, and modified in character by the supervention of numerous complications, each of which will give rise to morbid sensations and phenomena varying with the situation and function of the part affected.

Thus the febrile symptoms will be less marked, or may be altogether absent, or at least imperceptible; and the pain in the loins will be so reduced as to amount to little more than a sense of weight and fulness. The nausea assumes a more chronic disposition, being less frequently attended with vomiting, although in many cases the latter and chronic diarrhœa form leading complications, and by inducing exhaustion shorten the life of the individual. The proportion of albumen in the urine is less than in the acute stage, whilst the quantity of the latter fluid secreted, approaches to or may even exceed that of health, but is characterised by the small quantity of solids contained in it. According to Dr. Christison, the proportion of hæmatosine in the blood is greatly diminished in the advanced stages, a circumstance which he looks upon to be pathognomic of the nature, and even indicative of the progress of the disease: the blood is also deficient in albumen, and contains a great quantity of aqueous fluid, so that it coagulates faintly, and the clot is loose and gelatinous.

Mr. Robinson thinks that we should wait before we decide that the deficiency of hæmatosine in the blood is peculiarly characteristic of granular disease. May not hæmatosine be equally deficient in all cases of long-continued or excessive albuminous discharge? A question, in our opinion, very pertinent and reasonable.

Mr. Robinson makes some judicious remarks on the complications of granular disease, more particularly upon dropsy. He adds:—M. Rayer believes, that dropsy always occurs in granular disease of the kidney, or albuminous nephritis, as he terms it, and in that only; but Dr. Christison says, that dropsy does not always exist in that disease, and that some have even supposed it to be occasionally present in nephritis, thus doing away with any value that might be attached to this symptom as a peculiarity of granular kidney. If the above views as to its origin are correct, it is evident that it may or may not occur in nephritis; but that when present it indicates a debilitated or deranged condition of the cutaneous circulation, and therefore will be more generally met with as a symptom of nephritis in those localities, where from a cold climate, a marshy or damp soil, or a proximity to large surfaces of water, the functions of the skin as a secreting organ are more or less impaired.

The third chapter is occupied with the consideration of the morbid appearances of granular disease and of nephritis. Those of the acute stage of the former are quoted from Dr. Christison.

"The kidneys are flabby, friable, and much enlarged; they are darker and more vascular externally, and with points and star-like spots of ecchymosis; internally they are of a dark brownish red, or almost reddish black, gorged more or less with blood, which drops from a cut surface in unusual quantity; and they often present throughout their whole structure, but more especially in their cortical portion, lines and specks of a still darker colour and not easily removed by washing. Sometimes this congested state of the kidney prevails throughout the whole organ equally; at other times the cortical structure seems chiefly affected, and presents a more distinct and coarsely striated appearance than natural, probably from blood being injected or extravasated in lines into the fundamental cellular tissue. The cortical structure is almost always broader than in the healthy state, as if it were distended towards the circumference by its gorged condition.'

The appearances found in the kidneys of those who have suffered from anasarca and albuminous urine, consequent on scarlatina, are described as resembling very much those of the acute stage. In the article on this subject, in the Cyclopædia of Practical Medicine, the same author remarks that a granular deposit is present even in these acute cases, though it cannot at once be distinguished from the surrounding structure, as it is of the same colour, but may be recognised when the kidney is injected, as the fluid does not enter the granules." 30.

Such appearances might well be occasioned by acute congestion. Mr. Robinson produced this in an experiment. He cut down to and exposed one kidney in a rabbit and tied the renal vein, returning the organ immediately into its natural position with the artery uninjured. The animal died about fifteen hours afterwards, and on examination the kidney in question was the only organ found affected; it was intensely congested and very much enlarged, being nearly twice the bulk and weight of the healthy organ; of a dark red or blackish colour, in some places soft and easily torn, and the granular appearance on its surface more distinct than natural, as if from enlargement of the granules. On making a section it was throughout intensely gorged with blood, which seemed infiltrated into the cellular tissue of the organ: there was a little bloody urine in the bladder which contained two or three minute coagula, and became nearly a solid mass on applying heat. This experiment was repeated with precisely the same results as to the condition of the kidney, but from the ureter having been divided and tied, the urine secreted could not be examined. Perhaps the value of any conclusions drawn from these facts is increased from the circumstance of M. Rayer and Dr. Osborne having found fibrinous clots filling the renal veins in those who had died in the acute stage, an occurrence which shows that a very imperfect and retarded, if not a completely obstructed, circulation through those vessels had existed during life.

The granular appearance of the exterior was also more distinct in the congested kidney, arising from the distention of the granules naturally existing there. M. Rayer has likewise observed the Malpighian corpuscles to be congested in this acute form, and as they are composed chiefly of minute arteries, does it not seem very probable that the granules which Dr. Christison failed to inject (possibly from the renal *artery*), were merely these bodies enlarged from the distention of their constituent vessels in which the blood had coagulated, and from which, owing to their minuteness, it could not be washed out, preventing by its presence the entrance of the injected fluid into the granules?

After quoting Dr. Christison's summary of the morbid appearances of the chronic form of the complaint, Mr. Robinson avails himself of the arrangement of Dr. Bright, which adopts two leading varieties. In one, the cortical substance is made up of granules of various size and shades of colour, ranging from red to purple and yellow, the kidney being generally somewhat diminished and indurated; in the other, the organ is usually somewhat enlarged in bulk, its cortical substance consisting also of granules, but a flaky white interstitial deposit exists between them, masking their presence and rendering the structure apparently more homogeneous: after maceration, however, the interstitial deposit being washed away, the granules, as in the former case, are found to constitute the greater part of the cortical substance. So that the chief points of distinction between the two varieties are the greater induration and contraction in the one case, the granules being in close apposition with each other; whilst, in the second form, the size of the organ is somewhat increased, and the cellular tissue between the granules is replaced by what Mr. Robinson considers ordinary albuminous effusion.

The granules are looked upon by him as the Malpighian corpuscles in a state of congestion; and the following is his explanation of the two varieties in question.

"In both I suppose a degree of congestion to have existed for a sufficient length of time to produce enlargement of the Malpighian corpuscles, by the coagulation of the blood contained in their distended vessels: their various shades of colour are caused by the imperfect absorption of the colouring matter of the blood passing through all the intermediate tints from red to white as in other situations. But, in the first case, from the contraction and induration I should feel disposed to consider the disease as one of long continuance, and from the absence of any interstitial deposit it appears highly probable that a sufficient degree of inflammation, to produce albuminous effusion, had never existed in the organ, but that from some slight obstruction, retardation of the circulation through, and consequent fibrinous deposition in, the vessels of these corpuscles had gone on slowly and gradually to the destruction of the surrounding healthy tissue by absorption from their pressure. In the second form, the cause having been more intense in its action, and the examination of the organ having probably taken place at an earlier period, the appearances are very different. The congestion has been so great as not only to cause enlargement of the granules, but also to induce albuminous effusion into the intergranular cellular tissue, and of course the extent of this effusion will vary in different cases. This form may be considered as holding an intermediate place between the former or more chronic, and the acute stage first described; it may therefore be termed subacute nephritis. Hence a practical division of cases of nephritis, founded on their morbid appearances, into three forms or degrees. 1. The acute, in which the engorgement of the vessels has been so great as to cause their rupture, and sanguineous infiltration of the organ. 2. The subacute, or that accompanied with more or less interstitial albuminous deposit; and, 3. The chronic, in which the congestion has never been sufficient to produce any permanent kind of effusion. Suppuration may supervene on any of these forms, but in general only occurs in the two former." 40.

Mr. Robinson relates an experiment which we need not quote, as it is rather ingenious than conclusive. The fourth chapter is on the Causes of Granular Kidney, and asserts their identity with those of nephritis. The fifth chapter is dedicated to the consideration of the albuminous condition of the urine. Mr. Robinson notices the hypotheses that have been advanced to account for its occurrence. After some observations of rather a speculative character upon secretion, the gist of which is to shew that the blood contains the materials of the secretions as well as of the excretions, Mr. Robinson thinks himself justified in saying, that *albuminous effusion* is always the consequence of a congested or distended state of the capillaries of the part, and that in a healthy condition of the blood the *proportion* of albumen in the effused fluid may be considered as commensurate with the *degree* of that congestion.

The arguments by which this idea are backed must be deemed rather hypothetical, indeed fanciful, and we need not, therefore, insist upon them. He deduces from them this rule—That the presence of albumen in the urine is produced by, and its proportional quantity is in a direct ratio to, the degree of congestion of the capillaries of the kidney, from whatsoever cause that congestion may arise.

Mr. Robinson applies his rule to the explanation of albuminuria from other causes than granular disease. Thus, he remarks, albuminous urine has been met with by Solon in dropsy and obliteration of the cortical portion by cysts: by Rayer in three cases, one of hæmaturia with cancer and calculus in the kidney, another of true tubercular disease of it, and the third of inflammation of that organ and the bladder; by Christison in cerebriform disease; and by Syme in strumous disease. These cases are mentioned in Dr. Christison's work as well authenticated, and it seems to Mr. R. that they are all explicable by the above rule, for each morbid deposit, supposing it to act simply as foreign matter in occupying so much space, is adequate to the production of albuminous urine; but when, in addition to this, it is remembered that by its irritating action more or less inflammation of the contiguous portion or of the whole organ must be consequent on its continued presence, these two causes of obstruction to the renal circulation seem to me quite sufficient to account for that effect.

There is another class of cases in which, from various causes, the circulation through the abdominal and renal veins becomes more or less obstructed, and thus sometimes spontaneously, perhaps more frequently from the conjoined effect of accidental determination to the kidneys, a sufficient degree of congestion of these organs is produced to cause the temporary appearance of albumen in the urine. Thus it has been observed in cases of peritonitis (probably affecting chiefly that portion adjacent to the kidneys) by Nystel and others; in pregnancy by Rayer; during the crisis of fever, certain cutaneous affections, and some inflammatory diseases, (particularly pneumonia), by Solon; in hypertrophy of the heart, with valvular obstruction by M. Forget; in chronic inflammation of the liver by Graves; in phthisis, sometimes slightly in diabetic urine, and in a few other cases, in all of which, either from suspension of the cutaneous function and consequent increased action of the kidneys, or from a direct obstruction to the venous circulation in which the renal veins participated, some degree of congestion in these organs may naturally be supposed to have existed.

Mr. Robinson next alludes to the exceptions to the rule insisted on. They may, he observes, be briefly stated to be comprised in those cases in which pus or other albuminous matter is suspended in the urine from inflammation of some part of the genito-urinary mucous membrane. Thus it has been found more or less albuminous in cases of old and severe stricture, in a bladder inflamed and irritated by a calculus, during the passage of a calculus down the ureter, and would probably present the same character if examined in the acute stage of gonorrhœa; but it must also be remembered that these affections will each ultimately tend to produce renal congestion and irritation, so that in old cases it will sometimes be a doubtful point to determine whether the albumen is derived from the inflamed mucous membrane or from the kidney.

As the pelvis of the kidney may be considered to form a part of that organ, as we can hardly suppose its lining membrane to be inflamed without affecting that of the tubuli uriniferi, and thus involving the contiguous vessels, and as the definition of this as a distinct disease must be very difficult, and would serve no practical end even if it were possible, the recognition of its existence as a separate affection under the name of pyelitis, as proposed by M. Rayer, does not seem required.

A diseased condition or unnatural fluidity or tenuity of the blood, as in scurvy, or that peculiar condition of the vessels which induces excessive hæmorrhage from slight causes, and which has been found hereditary in some instances, may each cause the presence of albumen or blood in the urine.

Mr. Robinson offers an explanation of the low specific gravity of the urine in granular disease. Urea, says he, is found in the blood: it would seem to be the product of a species of decomposition of animal matter going on during the circulation of that fluid, the quantity of it excreted from the system being greatest after the consumption of azotised food, and commensurate with the proportion of animal principles existing in the blood. Now, during the progress of chronic nephritis, it has been shown that the blood becomes impoverished, the albumen escaping from it in the urine being replaced by water, and the debility being still further increased from the impediment to nutrition, occasioned by the frequent complication of vomiting and diarrhœa.

The substitution of water for the important principle, albumen, must necessarily diminish the quantity of those azotised matters formed in the blood by the decomposition or deterioration of its more animalised portion, and hence the deficiency of urea and the lithates in this disease. Viewed in this light, the low specific gravity of the urine cannot be considered as indicating more than an impoverished condition of the blood, which state we know may also occur in many other diseases, and hence any value which might otherwise attach to this symptom, as diagnostic of this particular affection, must be rendered doubtful till future experiments shall decide as to whether a similar state of the urine does not accompany the same condition of the blood in every disease in which the latter is present, as after long continued suppurative or other albuminous discharges.

The sixth Chapter, the concluding one, contains some practical deductions from the views that have been taken.

The first object is to determine whether albumen is present or not. The next is to assign it to its right cause, and by examination of the condition of the urethra and bladder, to endeavour to determine that cause. The absence, observes our author, of any adequate cause in the urethra or bladder being established, we may conclude that the albumen present in the urine arises from, and consequently indicates the existence of, a certain degree of congestion in one or both kidneys. By frequently examining the proportion of albumen in the urine, any increase or diminution in the intensity of the renal congestion can be at once detected, and the efficacy of any remedies tested. This simple examination of the urine will conduct the inquiry so far, and if any considerable decrease in the amount of solids contained in it be found to continue permanently, then the probability will be that from some cause or other the blood is in an impoverished condition; and here any assistance to be gained from ascertaining the constitution of the urine would seem to stop.

The causes of the affection must be ascertained by a careful examination of the symptoms and concurrent circumstances. Thus, remarks our author, whenever anasarca is found to co-exist, the possibility of the disease having been primarily induced by an impaired state of the skin will naturally suggest itself, and enquiries will be made with the view of learning whether from occupation or accident, or from the pre-existence of some exanthematous or other disease, such a disorder of the cutaneous circulation as would arrest its healthy function had not been produced. If the patient has been for a length of time affected with diseased heart or liver, or is in the advanced stage of phthisis, or if a tumor is situated near the course of the large venous trunks of the abdomen, and if the urine indicates only a slight degree of congestion in the kidneys, the inference will be that the latter affection is the consequence of obstruction to the return of blood through the renal veins from the existence of one of the former diseases. The age and appearance of the patient, and the simultaneous affection of the lung or other organs, will enable us to form an opinion as to the possibility of the renal congestion being kept up by scrofulous or tuberculous deposit in the organ; and if calculous matter has been passed with the urine at any former period, or if the present composition of that fluid is such as to strengthen the idea, we may, in the absence of any other adequate appreciable cause, attribute the albuminous urine and other signs of nephritis to the lodgment of one or more calculi in the kidney.

Mr. Robinson thinks that if we agree to his view, the prognosis will be rather more favourable than it has been. And it will of course give a tone to the treatment. Thus, in the acute form, repeated bleeding, general and local, active cathartics, diaphoretics the most direct and powerful, such as Dover's powder, antimonials and the warm or vapour bath, and afterwards counter-irritants, have been found most effectually to relieve the symptoms and diminish the intensity and danger of the attack. In chronic nephritis a less vigorous course of treatment, modified according to circumstances, but based on the same principles, is recognised as the only one from which beneficial results can be expected.

Mr. Robinson alludes to the vexata quæstio of diuretics. "If," says he, "the explanation which I have ventured to suggest, as to the immediate cause of the albuminous deposit in the kidney and also of albuminous urine, be correct, then all stimulating diuretics are decidedly contra-indicated so long as the continued existence of that cause is manifested. It even seems to me quite possible that large doses of diuretics given in the advanced stages of the disease, when the function of both kidneys has to be performed by a minute remaining portion of secreting tissue, may materially hasten the fatal termination by increasing the degree of renal congestion. Any arguments derived from an apparent increase of the solids discharged from the system in the urine under the use of diuretics must be more or less subject to fallacy, as it has been observed by Woehler, that all substances acting as diuretics contain principles which are themselves excreted by the kidneys.

When, therefore, diuretics are given at all, the condition of the kidney should be previously ascertained by examining the proportion of albumen in the urine, and if it is found in such minute quantity as to indicate a very slight degree of distention of the renal vessels, then the stimulus of very small doses of diuretics might perhaps cause the relaxed capillaries to recover their tone or contractility more quickly, and thus facilitate the progress of cure. If, however, diuretics are found to increase the proportion of albumen in the urine much, they should either be given in smaller doses and less frequently, or their use may be suspended for a time; and probably they should never be employed till after depletory measures have been resorted to for the purpose of reducing that increased arterial action on which the active congestion of the kidneys depends."

Blisters are naturally reprobated, and the employment of mercury, in the acute form, and in healthy persons, rather recommended.

The pamphlet before us, if tinctured by too much speculation to suit the practical spirit of the day, is by no means deficient in merit, and we have given the views of its author at some length because they appear to be in many respects just.

